# Effects of Varying Degrees of Intermittent Hypoxia on Proinflammatory Cytokines and Adipokines in Rats and 3T3-L1 Adipocytes

**DOI:** 10.1371/journal.pone.0086326

**Published:** 2014-01-21

**Authors:** Qing He, Qing-chan Yang, Qin Zhou, Hui Zhu, Wen-yan Niu, Jing Feng, Yan Wang, Jie Cao, Bao-yuan Chen

**Affiliations:** 1 Department of Endocrinology, Tianjin Medical University General Hospital, Tianjin, China; 2 Respiratory Department of Tianjin Medical University General Hospital, Tianjin, China; 3 Immunology Department, Tianjin Medical University, Tianjin, China; University Heart Center Freiburg, Germany

## Abstract

**Objectives:**

Intermittent hypoxia (IH), resulted from recurring episodes of upper airway obstruction, is the hallmark feature and the most important pathophysiologic pathway of obstructive sleep apnea (OSA). IH is believed to be the most important factor causing systemic inflammation. Studies suggest that insulin resistance (IR) is positively associated with OSA. In this study, we hypothesized that the recurrence of IH might result in cellular and systemic inflammation, which was manifested through the levels of proinflammatory cytokines and adipokines after IH exposure, and because IR is linked with inflammation tightly, this inflammatory situation may implicate an IR status.

**Methods:**

We developed an IH 3T3-L1 adipocyte and rat model respectively, recapitulating the nocturnal oxygen profile in OSA. In IH cells, nuclear factor kappa B (NF-κB) DNA binding reactions, hypoxia-inducible factor-1α (HIF-1α), glucose transporter-1 (Glut-1), necrosis factor alpha (TNF-α), interleukin (IL) -6, leptin, adiponectin mRNA transcriptional activities and protein expressions were measured. In IH rats, blood glucose, insulin, TNF-α, IL-6, leptin and adiponectin levels were analyzed.

**Results:**

The insulin and blood glucose levels in rats and NF-κB DNA binding activities in cells had significantly statistical results described as severe IH>moderate IH>mild IH>sustained hypoxia>control. The mRNA and protein levels of HIF-1α and Glut-1 in severe IH group were the highest. In cellular and animal models, both the mRNA and protein levels of TNF-α, IL-6 and leptin were the highest in severe IH group, when the lowest in severe IH group for adiponectin.

**Conclusions:**

Oxidative stress and the release of pro-inflammatory cytokines/adipokines, which are the systemic inflammatory markers, are associated with IH closely and are proportional to the severity of IH. Because IR and glucose intolerance are linked with inflammation tightly, our results may implicate the clinical relationships between OSA and IR.

## Introduction

Obstructive sleep apnea (OSA) is a common condition characterized by repeated episodes of upper airway obstruction which result in interruptions of breathing during sleep, recurring episodes of hypoxemia, sleep fragmentation, and daytime sleepiness. OSA affects 3%∼7% of adult men, 2%∼5% of adult women [1]–[3], and up to 4% of children [4]. OSA is associated with complications in different organ systems, such as cardiovascular morbidities, hypertension, obesity, dyslipidemia, insulin resistance (IR) [5], [6], diabetes [7] and metabolic syndrome [8], [9]. Although the influence of obesity likely exaggerates this risk, recent clinical studies suggest that IR and glucose intolerance are positively associated with OSA, independent of the degree of obesity [10].

Growing evidence from cellular, tissular and animal models of OSA shows that intermittent hypoxia (IH), resulted from recurring episodes of upper airway obstruction, the hallmark feature and the most important pathophysiologic pathway of OSA, is believed to be the most important factor causing systemic inflammation [11]–[12] which may play the key role in the development and progression of metabolic dysfunction [13]–[17]. Up until now, the precise mechanisms through which IH induces metabolic disturbances are still poorly understood [18]. Potential mechanisms may include hypoxia per se, sympathetic activation, and resultant systemic inflammation, involving the activation of inhibitory kappa B kinase (IκK)/inhibitory kappa B (IκB)/nuclear factor kappa B (NF-κB) pathway, disruption of hypothalamic-pituitary-adrenal axis, systemic catecholamine-mediated lipolysis and lipotoxicity, hepatic transcriptional upregulation of lipid synthesis, impaired lipid clearance, and the out-of-balance of glucose and insulin-regulating hormones produced by adipose tissue (adipokines), such as such as interleukin (IL) -6, tumor necrosis factor alpha (TNF-α) [16], leptin and adiponectin [8], [19], [20]. In addition, the chronic IH from OSA represses the expression of key genes regulating biosynthesis of pancreatic proinsulin convertases with a resultant progressive decrease in their catalytic activity. The long-term hypoxic damage to pancreatic β-cells may also contribute to progression of glucose dysregulation in patients with untreated OSA over time [21].

During the past 10 years, the view that adipose tissue is mainly a depot organ has profoundly evolved. The adipocyte is now considered as one of the most important mediators in metabolic and inflammatory regulation [22]. It releases a large number of classical adipokines, such as TNF-α, IL-6, leptin and adiponectin, and some newly discovered adipokines, such as tissue inhibitor of metalloproteinases-1 and monocyte chemotactic protein-1 [23]. Inflammation occurred in adipose tissue and cell is thought to play a key role in the development of the metabolic syndrome, type 2 diabetes and cardiovascular disease [24].

Leptin is one of the most important adipose-derived hormones that plays a key role in regulating energy intake and expenditure, including appetite and hunger, metabolism, and behavior. Circulating concentrations of leptin are proportional to the degree of IR [25]–[27]. Adiponectin, a cytokine produced in white adipose tissues, is a protein hormone that modulates a number of metabolic processes, including glucose regulation and fatty acid oxidation [28]. A reduction in adiponectin contributes to IR. Serum levels of adiponectin correlate positively with insulin sensitivity, and adiponectin also has anti-inflammatory or protective effects for vasculature system [26].

In this study, we developed an IH 3T3-L1 adipocyte and rat model respectively, recapitulating the nocturnal oxygen profile in OSA. And then in our cellular and animal models, we investigated the probable relationships between IH, which was utilized with varying degrees, and cellular and systemic inflammation. We hypothesized that the recurrence of IH and the following reoxygenation (ROX) might result in cellular and systemic inflammation, which was manifested through the levels of proinflammatory cytokines and adipokines after IH exposure, and because IR and glucose intolerance are linked with inflammation tightly according to widely accepted previous studies, this inflammatory situation may implicate an IR status through the levels of leptin, adiponectin and some other mediators indirectly in 3T3-L1 adipocyte culture medium and rat model and through blood glucose and insulin levels in rat plasma.

## Methods

### Animal Exposure to IH

To minimize the suffering of animals during the experiment and maximize the information from our animal study, we designed, analyzed and reported this research referring to the ARRIVE Guidelines [29], [30]. In every day, 9 AM to 5 PM is the sleeping time for Wistar rats, which were used in our study. According to our previous studies [31], [32], hypoxia treatment from 9 AM to 5 PM in mild to moderate severity will not stress Wistar rats, and they can be in a normal sleep status through out the exposure period. After anesthetization, the rats were sacrificed with exsanguination, which was also the process of blood sample collection, and even in preliminary experiments to get arterial blood gas (ABG) values, anesthetization was performed. Institutional Review Board of Tianjin Medical University General Hospital approved the ethical and methodological aspects of the investigation (TMU IRB Approving Number: EA-20120005). A total of 160 male Wistar rats weighing 180∼200 g at age of 8 weeks (provided by Model Animal Center of Radiological Medicine Research Institute, Chinese Academy of Medical Science, license No.: SCXK Tianjin 2006–0009) were divided into 5 groups of 32 each according to exposure conditions as follows: A. Rat control group (RC group, n = 32), sham IH exposure, intermittent normoxia; B. Rat sustained hypoxia group (RSH group, n = 32), sustained hypoxia exposure with air/N_2_ mixture, the concentration of O_2_ was about 10%; C. Rat IH group 1 (RIH-1 group, n = 32), IH exposure with the nadir O_2_ concentration of 5% in exposure chamber; D. Rat IH group 2 (RIH-2 group, n = 32), IH exposure with the nadir O_2_ concentration of 7.5% in exposure chamber; E. Rat IH group 3 (RIH-3 group, n = 32), IH exposure with the nadir O_2_ concentration of 10% in exposure chamber.

Model rats were exposed to IH for 8 weeks, 9 AM to 5 PM in every day. We used a hypoxia exposure device similar with those seen in our previous studies [11], [32]. Briefly, a gas control delivery system was designed to regulate the flow of nitrogen (N_2_, hypoxia phase) or clean air (air, ROX phase) alternatively into customized IH housing plexiglas chambers, 4 rats per chamber, to provide a designated IH or normoxia environment. A series of programmable solenoids and flow regulators altered the fractional concentration of inspired oxygen through software edited with Visual C^++^ computer language that controlled the durations of hypoxia (30 s) or ROX phase (90 s, normoxia), produced 30 cycles of alternations per minute. Gas flow was regulated and the environmental situation in the housing chamber for rats was monitored continuously with an O_2_ analyzer (CY-12C, Meicheng, China). During the hypoxia phase, the O_2_ concentration in the chamber was rapidly decreased to about 5% (RIH-1 group), 7.5% (RIH-2 group) or 10% (RIH-3 group) by adjusting the N_2_ flow rate ranging from about 5 L/min to 10 L/min. The O_2_ concentration was increased to a maximum of 21% by rapidly flushing the chamber with compressed clean air during ROX phase. The sham IH exposure rats (RC group) were exposed to a sham environment otherwise the same condition except the nitrogen source was changed to clean air source. To provide a sustained hypoxia environmental situation (RCH group), the air/N_2_ mixture, with the O_2_ concentration about 10%, was delivered continuously into the rat housing chamber for 8 weeks, 9 AM to 11 AM in every day. The chamber was equipped with a humidifier, thermostat and molecular sieve to maintain an inner temperature of ∼22°C, humidity of ∼45% and a relative germfree circumstance.

### 3T3-L1 adipocyte Differentiation

Murine preadipocytes (kindly donated by Prof. Amira Klip, The Hospital for sick children, Toronto, Canada) were maintained in Dulbecco’s modified Eagle’s medium (DMEM, 25 mmol/L glucose) containing 10% fetal bovine serum (FBS) in a 5% CO_2_ humidified atmosphere at 37°C. When the cells were subconfluenced at the culture bottle bottom, cell seeding was performed in 6-well plates. Cells were then cultured to confluence completely, followed by culturing for 48h when differentiation solution (0.5mmol/L 3-isobutyl-1-methylxanthine (IBMX), 0.25 µmol/L dexamethasone, 10mg/L insulin) was added to the DMEM containing 10% FBS. Additional culturing was performed for another 48 hours in medium containing 10 mg/L insulin. Subsequently, cells were induced to differentiate with DMEM containing 10% FBS during further 8 days to allow at least 90% of these cells to reach full differentiation before treatment. Differentiation was determined microscopically with the criteria that fat droplets were observed in cytoplasm. Differentiated 3T3-L1 adipocytes were resuspended with RPMI-1640 medium, and cell concentration was adjusted to 3×10^6^/mL. The cell cultures on 6-well plates with 1 mL per well (3×10^6^ cells/well) were then be treated as the following [33].

### Exposure of 3T3-L1 Adipocytes to IH

3T3-L1 adipocytes grown in culture plates were placed in plexiglas exposure chamber and were exposed to IH, sustained hypoxia (SH) or normoxia conditions for 6 hours per day during continuous 8 days. We developed a gas delivery system that permits the exposure of cell cultures to IH/ROX cycles, simulating the pattern of hypoxic episodes seen in recurrent apnea. Visual C^++^ computer language was used in coding a control-program (Breath-Simulating system 1.0) which regulated the delivery system as previously described [11], [32]. Briefly, a personal computer drove singlechip through serial port communicating-protocol, and with this system, solid relay was adjusted to modulate a gas resource system for controlling whether the gas mixture was given into the chamber or not. The gas mixture (Liu-fang Gas Limited Company, Tianjin, China) we used was consisted of O_2_ in particular concentration, 5% CO_2_ and balanced N_2_. The hypoxia gas mixture (O_2_ concentration of 5%, 7.5% or 10%, 5% CO_2_ and balanced N_2_, IH phase, 30 s) or normoxia gas mixture (21% O_2_, 5% CO_2_ and balanced N_2_, ROX phase, 90 s) flushed alternatively into the customized small IH housing chamber, to provide a designated IH or normoxia environment, produced 30 cycles of alternations per minute. The O_2_ concentration in the chamber was rapidly decreased to about 5% (CIH-1 group), 7.5% (CIH-2 group) or 10% (CIH-3 group) with different hypoxia gas mixture. The O_2_ concentration was increased to a maximum of 21% by rapidly flushing the chamber with normoxia gas mixture during ROX phase. The sham IH exposure (CC group) was exposed to a sham environment otherwise the same condition except the hypoxia gas mixture was changed to normoxia gas mixture. To provide a sustained hypoxia environmental situation (CSH group), a hypoxia gas mixture, with the O_2_ concentration about 10%, was delivered continuously into the IH chamber. The chamber was equipped with a humidifier, thermostat and molecular sieve to maintain an inner temperature of ∼37°C, humidity of ∼45% and a relative germfree circumstance. O_2_ levels in the culture media, ∼1mm above the cell layer, were sampled and monitored by oxygen electrode (Lazar, USA) and blood gas analyzer (AVL OMNI, Swiss), 6 samples per gas environment [34].

### Preliminary Experiments to Get Arterial Blood Gas (ABG) Values from Rats

To confirm the validity of our IH animal model, arterial blood gas (ABG) values from rats were measured in preliminary experiments. 2 rats were selected randomly from each group to obtain ABG data with the method used in our previous studies [31], [32]. Briefly, after light anesthetization (25% urethane, 4 mL/kg body weight intraperitoneally), the right femoral artery of selected rats was cannulated to obtain blood samples for monitoring ABG at anytime as necessary. The catheter was drawn out of the chamber through a small drilled hole. At different time points of the hypoxia cycle, arterial blood samples (0.7 mL each) were drawn and for each blood sample collected, ABG analysis included measurements of partial oxygen pressure (PaO_2_), partial carbon dioxide pressure (PaCO_2_) and arterial oxygen saturation (O_2_Sat) (AVL OMNI, Swiss).

### Sample Collection

In animal experiments, after 8 weeks of exposure, 8 rats from each group were measured for the body weight and then were anesthetized intraperitoneally with 3% pentobarbital at a dose of 30 mg/kg body weight. Fasting blood sample (8 mL ∼ 10 mL) from each rat was obtained from the right femoral artery. Plasma was isolated with centrifugation at 4000 rpm for 15min and frozen at −80°C. In cellular experiments, after exposure, the adipocyte medium (1 mL per well) was recovered and then reserved in −80°C for tests.

### Electrophoretic Mobility Shift Assay (EMSA) for NF-κB

3T3-L1 adipocytes harvested from each preparation were washed twice with ice-cold phosphate-buffered saline (PBS) and the cell suspension was transferred into a centrifuge tube. Cells were collected by centrifugation at 600×g for 5 min at 4°C, and then cytosol extraction reagent A (Active Motif, USA) was added to the cell pellet. The tube was vigorously vortexed to fully resuspend the cell pellet, and then after incubating the tube on ice, cytosol extraction reagent B was added into the tube. The tube was centrifuged for 5 min at maximum speed in a microcentrifuge (∼16,000×g) and the supernatant was removed. The insoluble (pellet) fraction produced in last step, which contains nuclei, was resuspended in icecold nuclear extraction reagent (Active Motif, USA). The tube was centrifuged at maximum speed (∼16,000×g) in a microcentrifuge and then the supernatant (nuclear extract) fraction was immediately transferred to a clean prechilled tube; all extracts were stored at −80°C until use. Nuclear protein concentrations were determined with bicinchoninic acid (BCA) protein assay for standardization. Nuclear factor κB (NF-κB) DNA binding reaction of nuclear extract was performed with nonradiolabeled electrophoretic mobility shift assay (EMSA, Weiao, Ningbo, China). Double-stranded oligonucleotide containing the consensus sequence of the binding site for NF-κB was 5′-AGTTGAGGGGACTTTCCCAGGC-3′, which was labeled with biotin. Reaction system: labeled probe 0.5 µL; 10X binding solution 1.0 µL; poly (dI:dC)(dI:dC) 1.0 µL; nuclear extract 0.4 µL–0.4 µg; total volume was regulated with distilled water till 10 µL.

### Western Blotting for Hypoxia-inducible Factor-1α (HIF-1α) and Glucose Transporter-1 (Glut-1) Protein Expression

Whole cell extracts were fractionated by 7.5% sulfate-poly-acrylamide gel electrophoresis (PAGE-SDS) gel electrophoresis and transferred to a polyvinylpyrrolidone difluoride membrane. The membranes were blocked by incubation for 1 h at room temperature with phosphate-buffered saline (PBS) containing 5% non-fat milk and 0.05% Tween 20 and then incubated with primary antibodies overnight at 4°C. HIF-1α or Glut-1 monoclonal antibody was applied at a dilution of 1∶500. Membranes were washed three times for 10 min in PBST before incubation for 1h at room temperature with horseradish peroxidase (HRP)-conjugated secondary antibodies. (goat anti-rabbit IgG, 1∶1,000 dilution; Boster Inc, Wuhan, China). After 6×5-min washes in PBST and 1×20-min wash in PBS, the membranes were incubated with enhanced chemiluminescence (ECL) for 1 min before exposure to the medical X-ray films. The membranes were reprobed with β-actin as a loading control.

### Quantitative Real-time RT-PCR

Total RNA was extracted from 3T3-L1 adipocytes with total RNA genes extraction kit according to manufacturer’s protocol. The cDNA synthesis from the isolated RNA was performed using a reverse transcriptional system. The primer sets are shown in Table 1. Quantitative real-time PCR was performed in a reaction containing cDNA and SYBR PCR master mix (Roche Group, Switzerland). Samples were analyzed with the ABI PRISM 7500 sequence detection system (Applied BioSystems,USA). All PCRs were performed in triplicate, and the specificity of the reaction was determined by melting curve analysis at the dissociation stage.

**Table 1:** The primer sets used for real-time RT-PCR.

Genes	Forward	Reverse
β-actin	GGCTGTATTCCCCTCCATCG	TGTACCGTAACAATGGTTGACC
HIF-1α	GGACGATGAACATCAAGTCAGCA	GGAATGGGTTCACAAATCAGCAC
Glut-1	CCATCCACCACACTCACCAC	AACTCTACGACTAGGACCCG
IL-6	CCGGAGAGGAGACTTCACAG	CAACACGTTACCGTTAAGAC
TNF-α	CGTCGTAGCAAACCACCAA	GGAACAGATGAGGGTCCAAGAG
Adiponectin	GCTCAGGATGCTACTGTTG	GAACCAGGATTCCCACTCT
Leptin	CCAGGATCAATGACATTTCACACAC	AGGTCATTGGCTATCTGCAGCAC

Note: HIF-1α, hypoxia-inducible factor-1α; Glut-1, glucose transporter-1; IL-6, interleukin-6; TNF-α, tumor necrosis factor α.

### ELISA

Enzyme-linked immunosorbent assay (ELISA) was performed with relevant kits (Invitrogen Inc, USA) for TNF-α, IL-6, leptin and adiponectin, both in rat plasma and in cell culture medium. Insulin levels in rat plasma were also measured with ELISA. Samples were run in duplicate according to manufacturer’s instructions. Average optical densities were used to calculate concentrations based on the standard curves generated with control peptides provided by the manufacturer.

### Biochemical Assay for Blood Glucose

Blood glucose levels of rats were measured by glucose oxidase-peroxidase (GOD-POD) reagents (Guigo Industrial Co., Ltd, Shanghai, China).

### Statistical Analysis

SPSS 17.0 (SPSS Inc., Chicago, IL) software package was used for statistical analysis and illustration. Preliminary ABG data was obtained and is displayed as a descriptive analysis. *Student’s t* test was used for comparing with baseline values and one way analysis of variance (*ANOVA*) was performed for whole difference among groups and *Bonferroni* post hoc multiple comparisons were used to evaluate differences between internal groups. Unless otherwise stated, values were reported as mean ± standard deviation (SD), and *P*<0.05 is considered statistically significant.

## Results

### ABG Results in Preliminary Animal Experiments

Table 2 summarizes the ABG results obtained from preliminary animal experiments. The values in this table are descriptive, not for comparing between groups. When rats in RIH-1 group, RIH-2 group and RIH-3 group were exposed to different levels of hypoxia situation, the minimum PO_2_ was dramatically reduced to 35.6±2.2844, 40.3±2.0371 and 48.8±0.3971 mmHg, respectively. The minimum PO_2_ was 37.4±2.1615 mmHg in rats of RSH group. In RC group, the minimum PO_2_ is 98.0±2.3353 mmHg as expected. There was no significant change in maximum PCO_2_ levels among groups.

**Table 2:** Descriptive ABG results from preliminary tests.

Groups	Hypoxia phase		ROX phase	
	Min PaO_2_ (mmHg)	Min O_2_Sat (%)	Max PaO_2_ (mmHg)	Max O_2_Sat (%)
RC	98.0±2.3353	97.8±0.2000	N/A	N/A
RSH	37.4±2.1615	64.3±1.1362	N/A	N/A
RIH-1	35.6±2.2844	60.1±1.7593	107.7±0.9894	97.6±2.1980
RIH-2	40.3±2.0371	70.7±1.0173	106.9±2.0871	97.4±2.9390
RIH-3	48.8±0.3971	78.8±1.5484	108.4±2.6095	97.5±2.5322

Note: ROX, reoxygenation; PaO**_2_**, partial oxygen pressure; PaO**_2_**, partial carbon dioxide pressure; O_2_Sat: arterial oxyhemoglobin saturation.

### PO_2_, PCO_2_ and PH Values in the Culture Media of 3T3-L1 Adipocytes

In cellular model, we collected the culture medium samples from about 1 mm above the cell layer and PO_2_, PCO_2_ and PH values were analyzed. The results were described as Table 3, which suggests that this cell model can provide hypoxia environment at the cellular level.

**Table 3:** The exposure environment at the cellular level provided by cell IH model.

Groups	Hypoxia phase			ROX phase		
	PO_2_mmHg	PCO_2_mmHg	pH	PO_2_mmHg	PCO_2_mmHg	pH
CC	75.82±1.5834	38.40±0.8544	7.33±0.0060	N/A	N/A	N/A
CSH	55.12±2.1371	37.96±1.0090	7.41±0.0051	N/A	N/A	N/A
CIH-1	55.48±0.9257	37.56±1.3686	7.40±0.0080	75.34±1.3353	38.42±0.8526	7.39±0.0116
CIH-2	60.21±0.2876	37.62±1.0982	7.40±0.0101	75.95±1.6383	38.28±0.3249	7.39±0.0174
CIH-3	68.52±2.2521	37.48±1.3461	7.40±0.0117	76.80±1.9118	38.22±1.4686	7.40±0.0145

Note: Note: ROX, reoxygenation; PO_2_, partial oxygen pressure; PCO_2_, partial carbon dioxide pressure.

### Body Weight

Body weight of rats was different among groups after 8 weeks of exposure (*F* = 456.00, *P = *0.000), when the weight in RIH-1 or RSH group was the lowest (all *P* values <0.05) and there was no statistical difference between RIH-1 and RSH (*P* = 0.017). Rats in RC group got the highest weight (all *P* values <0.05).

### Insulin and Blood Glucose Levels in Rats

**Figure 1:**
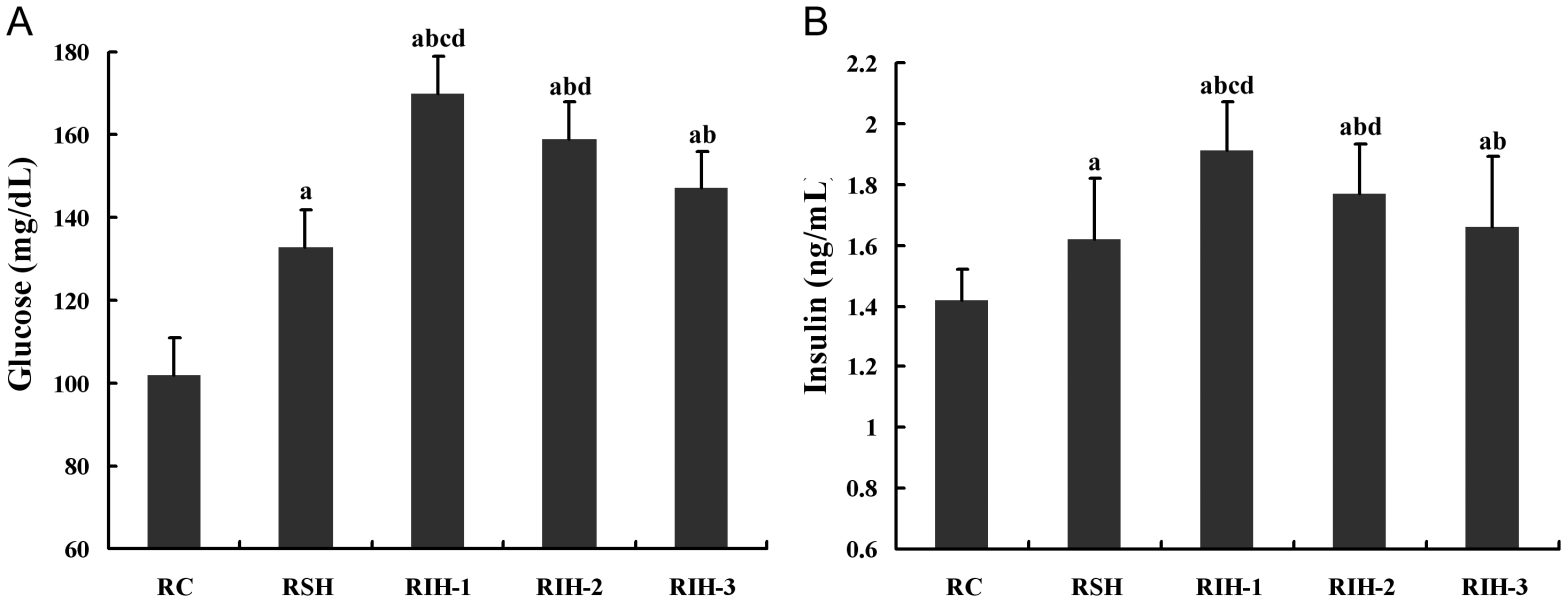
Insulin and blood glucose levels in rats. ^a^ *P*<0.05 compared with RC group; ^b^ *P*<0.05 compared with RSH; ^c^ *P*<0.05 compared with RIH-2,.^ d^ *P*<0.05 compared with RIH-3.

Insulin and blood glucose levels in rat plasma are presented in Figure 1. After 8 weeks of exposure, both the insulin and blood glucose levels in all groups were different statistically. There was significant difference in *Bonferroni* post hoc multiple comparisons (all *P* values <0.05) which indicated that at the end of 8-week treatment, both the insulin and blood glucose levels have significantly statistical results described as RIH-1> RIH-2> RIH-3> RSH>RC.

### EMSA Results of NF-κB DNA Binding Reactions in 3T3-L1 Adipocytes Nuclear Extracts

**Figure 2:**
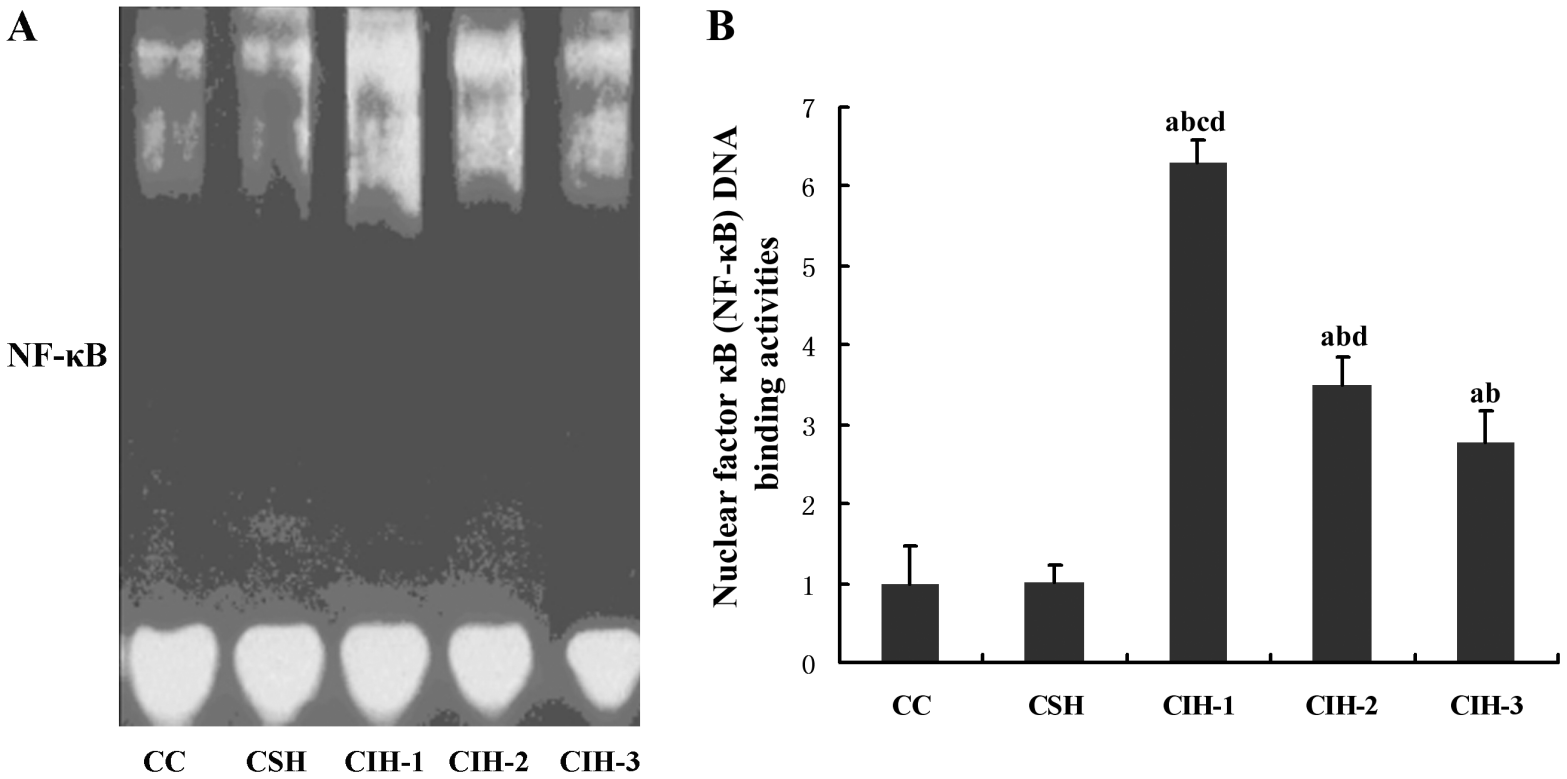
NF-κB DNA binding reaction of nuclear extract was performed with nonradiolabeled EMSA. ^a^ *P*<0.05 compared with CC group; ^b^ *P*<0.05 compared with CSH; ^c^ *P*<0.05 compared with CIH-2,.^ d^ *P*<0.05 compared with CIH-3.

NF-κB DNA binding reactions were significantly different among groups of cellular model after exposure (*F* = 447.02, *P* = 0.000). This activity in the CIH-1 group (6.298±0.275) was the highest and NF-κB DNA binding activities in CIH-2 group was higher than those in CIH-3, CSH or CC groups (all *P* values <0.05). Those levels were higher in RIH-3 group when compared with RSH or CC group (all *P* values <0.05), when there is no significant difference between CSH group and CC group (*P* = 1.000) (Figure 2).

### Effects of IH on mRNA and Protein Levels of HIF-1α and Glut-1 in Cellular Model

#### HIF-1α

**Figure 3:**
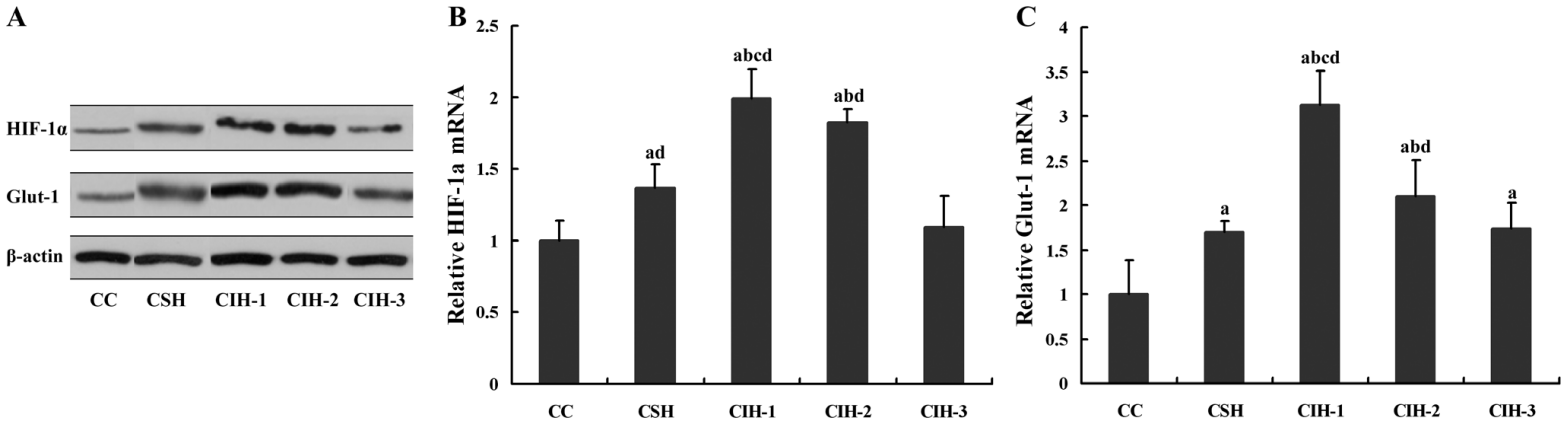
Effects of IH on mRNA and protein levels of HIF-1α and Glut-1 in cellular model. ^a^ *P*<0.05 compared with CC group; ^b^ *P*<0.05 compared with CSH; ^c^ *P*<0.05 compared with CIH-2, ^d^ *P*<0.05 compared with CIH-3. Note: HIF-1α, hypoxia-inducible factor-1α; Glut-1, glucose transporter-1.

Both the mRNA and protein levels of HIF-1α were different statistically among cellular groups (*F* = 7.312, *P* = 0.02 for mRNA). Those levels were the highest in CIH-1 group (all *P* values <0.05), and those levels in CIH-2 group were higher than those in CIH-3, CSH or CC group (all *P* values <0.05). Those levels were higher in RSH group when compared with CIH-3 or CC group (all *P* values <0.05), when there is no significant difference between CIH-3 and CC group (*P* = 0.083) (Figure 3).

#### Glut-1

Both the mRNA and protein levels of Glut-1 were different statistically among cellular groups (*F* = 24.636, *P = *0.000 for mRNA). Those levels were the highest in CIH-1 group (all *P* values <0.05), the lowest in CC group (all *P* values <0.05), and those levels in CIH-2 group were higher than those in CIH-3, CSH or CC group (all *P* values <0.05). There is no significant difference between CIH-3 and CSH group (*P* = 0.055) (Figure 3).

### Effects of IH on mRNA and Protein Levels of TNF-α and IL-6

**Figure 4:**
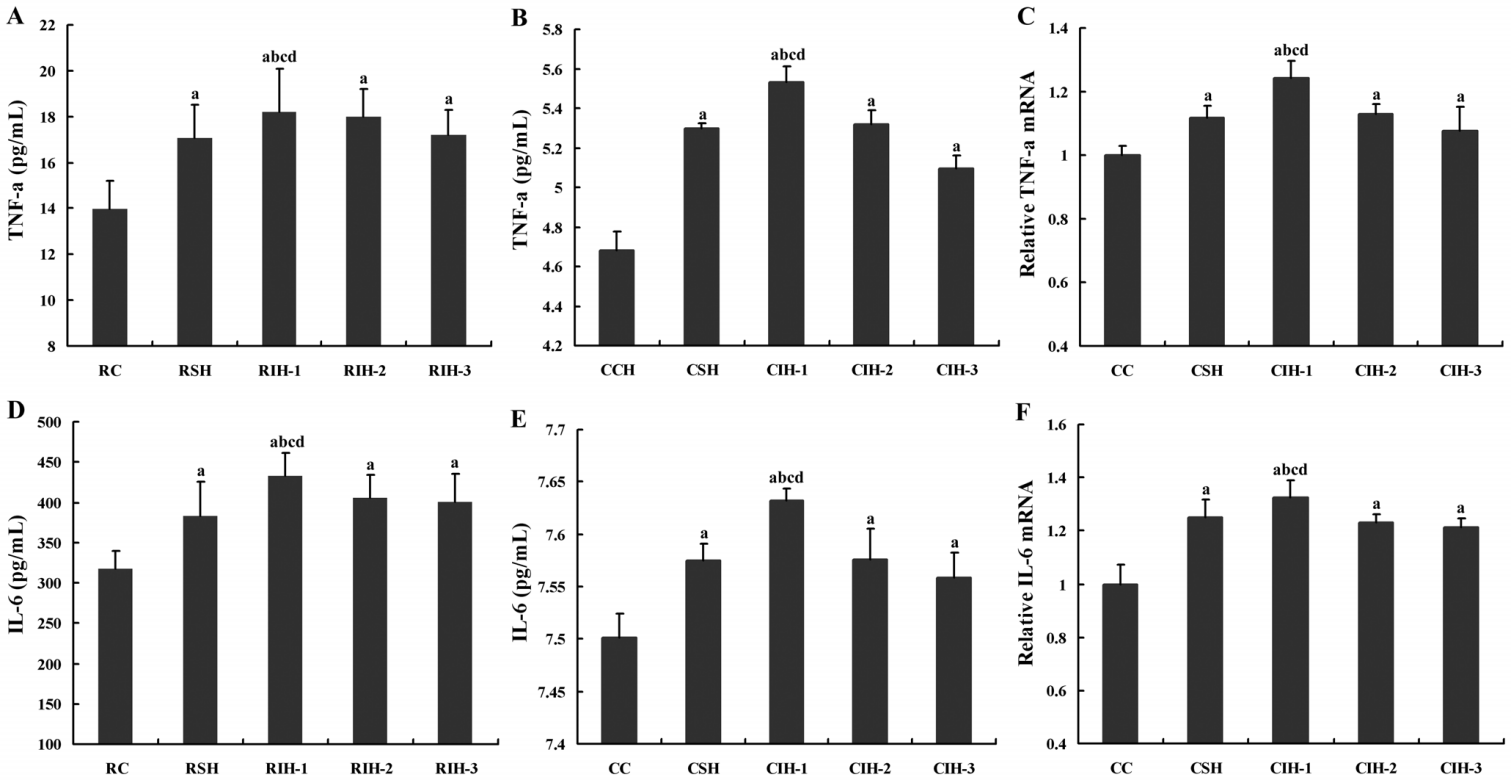
Effects of IH on mRNA and protein levels of TNF-α and IL-6. ^a^ *P*<0.05 compared with RC or CC group; ^b^ *P*<0.05 compared with RCH or CSH; ^c^ *P*<0.05 compared with RIH-2 or CIH-2,.^ d^ *P*<0.05 compared with RIH-3 or CIH-3. Note: IL-6, interleukin-6; TNF-α, tumor necrosis factor α.

In cellular and animal models, both the mRNA and protein levels of TNF-α and IL-6 were different statistically among groups (*F* = 14.736, *P*<0.05). Those levels are the highest in CIH-1/RIH-1 group (all *P* values <0.05), the lowest in CC/RC group (all *P* values <0.05), when there is no significant difference among CIH-2/RIH-2, CIH-3/RIH-2 and CSH/RSH group (all *P* values <0.05) (Figure 4).

### Effects of IH on mRNA and Protein Levels of Leptin and Adiponectin

**Figure 5:**
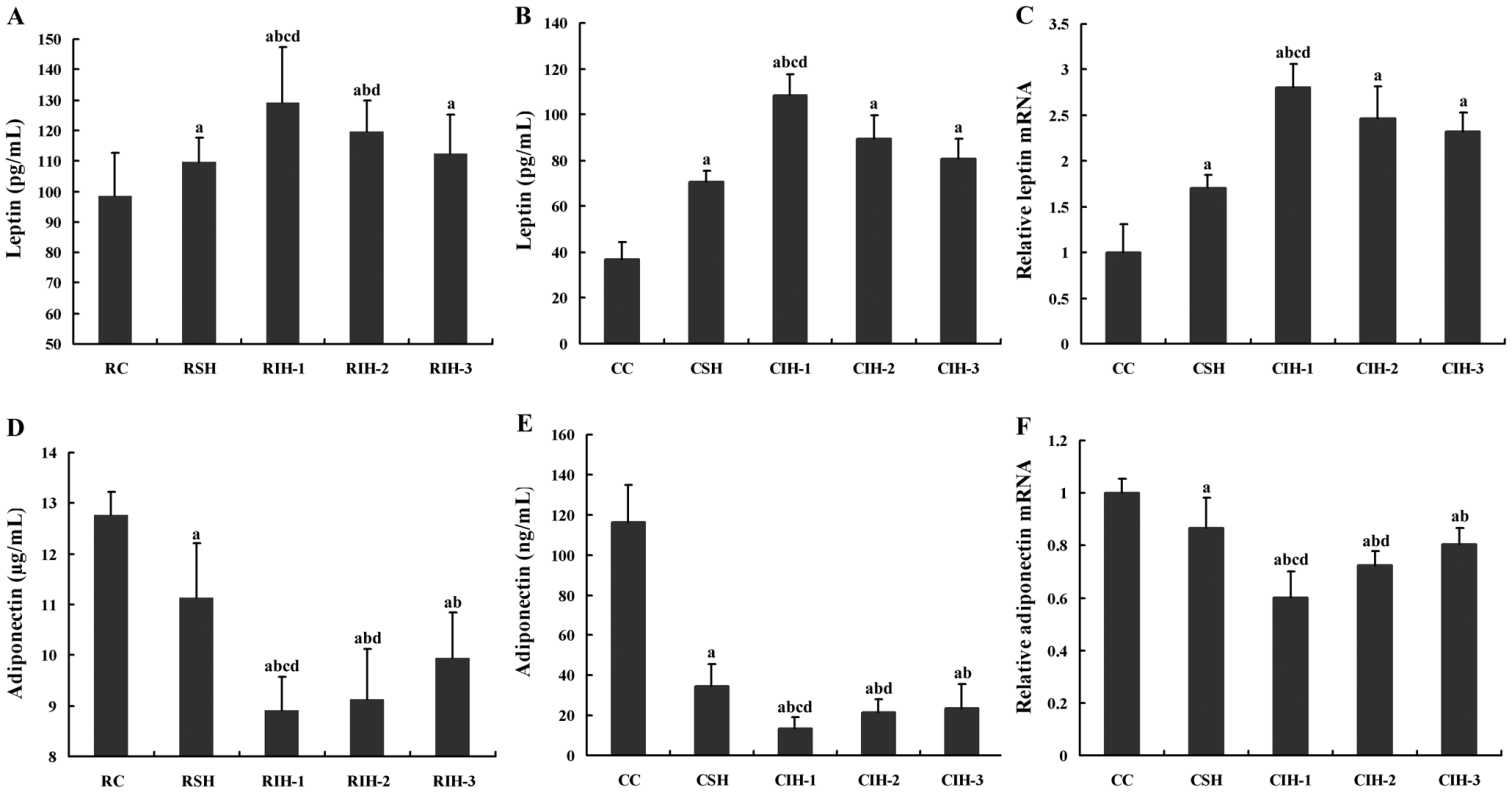
Effects of IH on mRNA and protein levels of Leptin and Adiponectin. ^a^ *P*<0.05 compared with RC or CC group; ^b^ *P*<0.05 compared with RCH or CSH; ^c^ *P*<0.05 compared with RIH-2 or CIH-2,.^ d^ *P*<0.05 compared with RIH-3 or CIH-3.

In cellular and animal models, both the mRNA and protein levels of leptin and adiponectin were different statistically among groups (*F* = 27.499, *P*<0.05). There was significant difference in *Bonferroni* post hoc multiple comparisons (all *P* values <0.05) which indicated that at the end of treatment, the leptin levels have significantly statistical results described as RIH-1> RIH-2> RIH-3> RSH>RC and CIH-1> CIH-2> CIH-3> CSH>CC, when the adiponectin levels have significantly statistical results described as RIH-1< RIH-2< RIH-3< RSH<RC and CIH-1< CIH-2< CIH-3< CSH<CC (Figure 5).

## Discussion

It has been suggested that there is a close association between inflammation and IR [26]. IR is one of the main pathophysiological characteristics of metabolic syndrome, and abnormalities in insulin-glucose metabolism have been repeatedly shown in OSA patients, especially when OSA is severe [24], [35]. Recurrent obstructive events from OSA result in alternating cycles of IH/ROX, and rapid reoxygenation of transiently ischemic tissues may lead to tissue injury and release of reactive oxygen species (ROS), the culprit of oxidative stress. These ROS molecules are key activators for inflammatory pathways [24]. IH and resultant oxidative stress have been proposed to be a pathogenetic link between OSA and disturbance of metabolism homeostasis [36]. One attractive possibility is that ROS-induced IR is mediated by Jun N-terminal kinase (JNK), which can be activated by oxidative stress [37]. It is suggested that the inhibition of JNK activity, through genetic knockout or a certain inhibitory peptide, improved insulin sensitivity in mice [38]. The potential mechanism is TNF-α and IL-6 initiates signaling cascades that converge on the inhibitory kappa B kinase (IKK) complex via the c-JNK and NF-κB signaling pathways. IKK can directly inhibit insulin signaling by phosphorylating insulin receptor substrate-1 (IRS-1) on serine residues, contributing to the development of IR [39], [40].

It is now generally accepted that white adipose tissue is a key endocrine and secretory organ which releases various adipokines with links to inflammation and immunity. The paradigm shift in adipose tissue biology was initiated in 1994 by the discovery of leptin. Subsequently, a continually increasing range of proteins, peptides and other factors, collectively termed adipokines, have been described to be released from white adipocytes. The majority of these adipokines are linked to inflammation and their production is increased in obesity. Increased expression and secretion of adipokines is a marker of chronic inflammation in adipose tissue [24]. In this study, we exposed adipocytes and rats to IH/ROX cycles, explored the inflammatory situations in cellular and animal systemic levels, confirmed the links between IH and inflammation. And because IR and glucose intolerance are linked with inflammation tightly according to widely accepted previous studies [24], [26], we implicated the clinical relationships between OSA and IR.

We demonstrated that plasma insulin and glucose levels in IH exposure rat groups (RIH-1, RIH-2 and RIH-2 group) are higher than RC and RSH group, when RIH-1 group shown the highest levels. In a clinical study, patients with OSA had significantly higher fasting plasma insulin levels than those in body mass index (BMI)-matched obese controls, and there was a modest relationship between apnea hyponea index (AHI) and fasting insulin levels, but not fasting blood glucose levels [41]. There are several potential mechanisms of impaired insulin secretion and increased glucose levels during IH. IH activates the sympathetic nervous system, which is a potent stimulator of lipolysis resulting in the release of free fatty acid (FFA). FFA reduces insulin-mediated whole body glucose uptake within hours due to interruption of insulin signaling in skeletal muscle [42]. In addition, catecholamines from sympathetic activation of IH may directly stimulate the mobilization of glycogen and inhibit glucose uptake from muscle, stimulate secretion of glucagon, inhibit secretion of insulin, and increase gluconeogenesis in the liver [43]. IH also activates the hypothalamic-pituitary-adrenal axis [44]. The resulting release of corticosteroids has well-defined effects leading to IR [45], including an increase in lipolysis, inhibition of insulin-dependent translocation of Glut-4 to the cell surface in muscles, suppression of glycogen synthesis and increase in gluconeogenesis. IH increases leptin gene expression and circulating leptin protein levels. Leptin acts both centrally and peripherally, to inhibits insulin secretion while increasing glucose uptake [18]. In comparison with RC group, plasma insulin and glucose levels in RSH group are higher but no statistical significance was obtained. This result is not consistent with a previous in vivo study. In the study by Polotsky [9], long-term exposure to SH did not cause IR and resulted in a decrease in fasting blood glucose levels and the glucose tolerance was unchanged. The detailed reason for this controversy is not explicit and further research is needed.

HIF-1 is considered as one of the master regulators of oxygen homeostasis and plays a key role in the response to hypoxia in most tissues [24]. It is composed of HIF-1α (120 kDa) and aryl hydrocarbon receptor nuclear translocator (ARNT; also called HIF-1β) (94 kDa) subunits. HIF-1α and ARNT are constitutively expressed, when the formation of HIF-1 transcription factor in the nucleus depends on HIF-1α stabilization, which is O_2_-dependent [46]. HIF-1 is activated in hypoxia through a well-defined mechanism, resulting in increased expression of a number of genes encoding proteins such as erythropoietin (EPO), vascular endothelial growth factor (VEGF), and inducible nitricoxide synthase (iNOS) which increase tissue oxygenation. These factors allow an adaptation to hypoxia that is directed toward increasing tissue perfusion and oxygenation and hence overcoming the initial hypoxic insult [47]. Furthermore, during the adaptive response to hypoxia, the expression of several genes encoding glycolytic enzymes is recognized to increase. To sustain higher levels of glycolysis, there is a need for an increase in glucose uptake, and this could be aided by an increase in the level of Glut-1 expression in hypoxia. Glut-1 is a key facilitative glucose transporter in white adipocytes and mainly mediates glucose uptake [48], and it is also related to IR [49]. Glut-1 protein is located predominantly in the plasma membrane [50] and its gene is regulated by HIF-1α that can detect the hypoxia situation [49]. So, Glut-1 is one of the key components of the HIF-1α- mediated hypoxia response. Compared with HIF-1α, Glut-1 itself is a relatively stable protein and therefore induces a more sustained profile of response that is likely to persist in regions that experience hypoxia and subsequently undergo reoxygenation [51]. In cellular model, HIF-1α and Glut-1 levels increased significantly in cells exposed to CIH-1, CIH-2 or SH compared with CC group, when CIH-1 exposed cells had the highest levels. HIF-1α and Glut-1 mRNA levels were elevated with IH or SH exposure suggesting a transcriptional activation and an adaptive response to hypoxia situation. It is also implicated that the increased HIF-1α and Glut-1 levels in IH/OSA may contribute to the increasing of blood glucose and the development of IR clinically.

Rats and cells exposed to IH exhibited greater circulating levels of TNF-α and IL-6 as compared with the counterparts exposed to SH or normoxia, and we also observed higher levels of proinflammatory cytokines in SH groups than those in normoxia groups. These results suggest that IH may elicit more severe systemic inflammatory responses than SH and these proinflammatory responses culminated in severe IH exposed rats or cells. Parts of our findings are consistent with other reports of increased TNF-α and IL-6 levels in cells or rats subjected to IH [52], [53]. However, in study by Wang et al, authors found that the expression of TNF-α in human adipocytes was not altered after hypoxia exposure, and this gene might not seem to be hypoxia-sensitive [48]. The possible reason accounting for this discrepancy may be the subjects, the way or the time periods of IH were different from our experimental paradigm. Furthermore, TNF-α and IL-6 levels in CIH-1 group and RIH-1 group treated with 5% IH were the highest among groups. These results showed that oxidative stress and the release of pro-inflammatory cytokines/adipokines, which are the systemic inflammatory markers, are associated with IH and are “IH dose-dependent”.

The possible explanation for the increase of inflammatory cytokines is NF-κB pathway activation from IH exposure, which was confirmed in our study. NF-κB DNA binding reactions were significantly increased and those levels were the highest in CIH-1 group. Those results proposed that IH may initiate remarkable proinflammatory responses and these responses were proportional to the severity of IH. NF-κB pathway activation in IH exposure may be more evident than in SH. Similar results has been advised and the mechanisms were also ever proposed in one of our previous studies [52]. The feature that distinguishes IH from SH is the intervening periods of normoxia. In SH, the mitochondria consume almost all of the O_2_, and rapid stabilization of HIF-1 occurs, which leads to increased activation of the adaptive pathway [54]. In IH/ROX, the extent of hypoxia is not sufficient to allow HIF-1 stabilization; however, possibly through mitochondrial dysfunction induced by ROX, it results in oxidative stress and then inflammatory stimulation which activates NF-κB, with the downstream consequence of intensity-dependent activation of the inflammatory pathway that depends on NF-κB translocating into the nucleus [55], [56]. NF-κB is a well-accepted redox-sensitive transcription factor that participates in numerous pathological conditions including inflammatory processes and cell apoptosis. In resting cells, NF-κB is sequestered in cytoplasm by IκB which undergoes phosphorylation, ubiquitination, and degradation upon stimulation, leading to the translocation of NF-κB into nucleus and activates transcription of target genes, including inflammatory factor genes mainly [57]. The positive feedback regulation has been proposed for regulation of NF-κB activation. In exposure to IH, NF-κB is activated and stimulates increased production of circulating cytokines such as TNF-α and IL-6, which, in turn, activates signaling cascades to enhance NF-κB activation. The positive regulation contributes to amplification of the inflammatory responses [52], [58]. The precise mechanisms through which IH mediates NF-κB activation need further investigation. In addition, it has been shown previously that TNF-α stimulates secretion of IL-6 via a NF-κB-dependent pathway. However, the opposite regulation of TNF-α and IL-6 has also been demonstrated in a recent study in rats [59], where OSA caused an increase in serum corticosterone levels, which was associated with up-regulation of IL-6 in retroperitoneal adipose tissue and down-regulation of TNF-α in mesenteric adipose tissue [21]. Recently, it is reported that the inflammatory cytokines of TNF-α and IL-6 are elevated in sleep apnea and obesity and might play a role in the pathogenesis and pathological sequelae of both disorders. TNF-α correlates strongly with lipolysis, and this cytokine causes marked IR and stimulates leptin secretion, where the precise mechanisms also need further investigation [41].

Human OSA is associated with high levels of circulating leptin [17] and the increase in leptin occurred in proportion to severity of OSA [26]. Leptin was significantly increased in sleep apneas compared to levels in both BMI-matched obese and normal weight subjects [41] and a reduction of serum leptin levels was observed after weeks or months of continuous positive airway pressure (CPAP) treatment [26]. Leptin, a main cytokine coded by obesity gene and secreted by adipocytes, especially higher levels in obese patients [11], is essential, and it acts not only in the arcuate hypothalamus nucleus as a satiety signal, but also in the inflammatory and endothelial systems [60]. Leptin can act at the level of the pancreas to downregulate insulin gene transcription and insulin secretion and at the peripheral tissues to increase glucose uptake [9]. Leptin also has an immunomodulatory role and is both activated by proinflammatory mediators and works as a stimulant of proinflammatory cytokine production such as IL-6 and TNF-α [61]. Hypoxia is another leptin-inductive factor [11]. The report by Christian et al. suggests that, in lean mice, all types of hypoxia caused striking increases in serum leptin, 17-fold in infrequent IH, 9-fold in frequent IH, and 44-fold in SH [17]. In leptin-deficient obese mice, repeated exposure to IH (30s hypoxia alternating with 30s normoxia, 12 h/day) for 12 weeks led to a time dependent increase in fasting insulin level and deterioration in glucose tolerance and IR [26]. Our study showed that leptin levels both in IH groups and SH groups increased significantly compared with normoxia group. The increase in leptin occurred in proportion to severity of OSA in IH exposure groups. Leptin plays an important role in mitigating the metabolic disturbances that accompany IH. Thus, the elevation of leptin levels caused by the hypoxic stress induced by IH exposure may represent an important compensatory response that acts to minimize metabolic dysfunction [9]. However, in some previous reports, SH induced higher leptin levels than IH [17], [26], and in another previous study, 5-day IH exposure elevates circulating leptin levels, but a lower leptin level observed at the 4-week time point in this study [62], which are contradictory with our results. One of the possible explanations for this discrepancy may be related to different degrees and frequencies of IH utilized in various experiments. Leptin gene expression is regulated by HIF-1 and an increase in leptin may reflect the effect of HIF-1 on systemic inflammation from IH, which is another possible explanation for our data [63].

When majority of adipokines are linked to inflammation, the only known exception is adiponectin, a potent anti-inflammatory adipokine with insulin-sensitizing effects [24]. It has been confirmed to play a key role in maintaining energy homeostasis and regulating metabolism of glucose and fat [64]. Adiponectin participates in the ameliorating of IR. The mechanism is that adiponectin decreases circulating FFA by increasing fatty acid oxidation by skeletal muscle. This results in decreased triglyceride content in muscle that has been associated with improved insulin sensitivity. In addition to its effects on fuel homeostasis, adiponectin may have anti-inflammatory properties. Adiponectin inhibits myelomonocytic activity, phagocytic activity, TNF-α production by macrophages [65], NF-κB activation and hence the production of IL-6 and TNF-α. It also induces production of the anti-inflammatory IL-10. On the other hand, oxidative stress, TNF-α, and IL-6 inhibit adiponectin production, hence potentiating their effects [61]. Adiponectin secretion is known to be regulated at the adipocyte level and an earlier study has shown that SH alters adiponectin expression and secretion [10]. In another previous study, it was demonstrated that plasma levels of adiponectin was suppressed in subjects with severe OSA, independent of obesity [66]. However, studies on serum adiponectin levels in OSA are controversial [26]. In a study of 68 subjects with no known comorbidity undergoing sleep studies, OSA subjects actually had a higher level of adiponectin compared with BMI-matched non-OSA subjects [67]. In a case-control study of 28 other-wise healthy subjects with moderate OSA, McArdle et al. reported that there was no difference in adiponectin levels between OSA and BMI-matched non-OSA subjects [26]. Furthermore, the use of CPAP treatment for 2 to 3 months was reported to increase serum adiponectin levels significantly in OSA patients [68], but not in a randomized controlled study of diabetics with OSA [26]. In this study we demonstrated that IH caused a decrease in the total secreted adiponectin by adipocytes, and the decrease in adiponectin secretion was associated with a downregulation of adiponectin mRNA expression. This reduction, albeit modest, in adiponectin production in adipocytes and rats is consistent with a recent report in human adipocytes [48]. The potential mechanisms are that IH may activate NF-κB, inhibit histone deacetylase 3 (HDAC3) translocating into the nucleus, and prevent peroxisome proliferator-activated receptor γ (PPAR-γ)-dependent pathways, and it has been well documented that PPAR–γ activity also can be inhibited by TNF-α [69]. PPAR-γ is the most important transcription factor mediating the expression of adiponectin. The inhibition of PPAR-γ will decrease the synthesis and secretion of adiponectin.

In human, OSA repeatedly interrupts sleep and thereby makes adults sleepy during the day. This daytime sleepiness translates into inactivity and excess weight, and these symptoms correlate with OSA severity [70]. But in our study, after 8 weeks of exposure, the weight gain in RIH-1 or RSH group was the lowest. The possible reason accounting for this may be that hypoxia may affect the growth and development of these rats. And our IH rat model can not mimic the OSA patients completely, which is a shortcoming of our experiment. Though with a relatively light weight, RIH-1 group still show the highest levels in pro-inflammatory cytokines and adipokines. The links between IH and inflammation are confirmed. Oxidative stress and the release of pro-inflammatory cytokines/adipokines, which are the systemic inflammatory markers, are associated with IH closely and are proportional to the severity of IH. Because the close association between inflammation and IR has been generally accepted, the clinical relationships between OSA and IR are implicated.
